# Nintedanib reduces ventilation‐augmented bleomycin‐induced epithelial–mesenchymal transition and lung fibrosis through suppression of the Src pathway

**DOI:** 10.1111/jcmm.13206

**Published:** 2017-06-09

**Authors:** Li‐Fu Li, Kuo‐Chin Kao, Yung‐Yang Liu, Chang‐Wei Lin, Ning‐Hung Chen, Chung‐Shu Lee, Chih‐Wei Wang, Cheng‐Ta Yang

**Affiliations:** ^1^ Department of Internal Medicine Division of Pulmonary and Critical Care Medicine Chang Gung Memorial Hospital and Chang Gung University Taoyuan Taiwan; ^2^ Department of Respiratory Therapy Chang Gung Memorial Hospital Taoyuan Taiwan; ^3^ Chest Department Taipei Veterans General Hospital Taipei Taiwan; ^4^ Institutes of Clinical Medicine School of Medicine National Yang‐Ming University Taipei Taiwan; ^5^ Department of Pathology Chang Gung Memorial Hospital Taoyuan Taiwan

**Keywords:** epithelial–mesenchymal transition, nintedanib, Src, pulmonary fibrosis, ventilator‐induced lung injury

## Abstract

Mechanical ventilation (MV) used in patients with acute respiratory distress syndrome (ARDS) can increase lung inflammation and pulmonary fibrogenesis. Src is crucial in mediating the transforming growth factor (TGF)‐β1‐induced epithelial–mesenchymal transition (EMT) during the fibroproliferative phase of ARDS. Nintedanib, a multitargeted tyrosine kinase inhibitor that directly blocks Src, has been approved for the treatment of idiopathic pulmonary fibrosis. The mechanisms regulating interactions among MV, EMT and Src remain unclear. In this study, we suggested hypothesized that nintedanib can suppress MV‐augmented bleomycin‐induced EMT and pulmonary fibrosis by inhibiting the Src pathway. Five days after administrating bleomycin to mimic acute lung injury (ALI), C57BL/6 mice, either wild‐type or Src‐deficient were exposed to low tidal volume (V_T_) (6 ml/kg) or high V_T_ (30 ml/kg) MV with room air for 5 hrs. Oral nintedanib was administered once daily in doses of 30, 60 and 100 mg/kg for 5 days before MV. Non‐ventilated mice were used as control groups. Following bleomycin exposure in wild‐type mice, high V_T_ MV induced substantial increases in microvascular permeability, TGF‐β1, malondialdehyde, Masson's trichrome staining, collagen 1a1 gene expression, EMT (identified by colocalization of increased staining of α‐smooth muscle actin and decreased staining of E‐cadherin) and alveolar epithelial apoptosis (*P *<* *0.05). Oral nintedanib, which simulated genetic downregulation of Src signalling using Src‐deficient mice, dampened the MV‐augmented profibrotic mediators, EMT profile, epithelial apoptotic cell death and pathologic fibrotic scores (*P *<* *0.05). Our data indicate that nintedanib reduces high V_T_ MV‐augmented EMT and pulmonary fibrosis after bleomycin‐induced ALI, partly by inhibiting the Src pathway.

## Introduction

ARDS is a severely debilitating disease with a high mortality rate of 27–45% 1, 2. MV is required to save the lives of patients with ARDS. However, despite widespread application of lung‐protective ventilation strategies, ventilator‐induced lung injury (VILI) continues to occur. VILI is characterized by an initial epithelial injury followed by or combined with fibroproliferative activity in the lungs of patients who experience ARDS 1, 2, 3, 4, 5. Pathologic fibroproliferation appears to play a pivotal role in both short‐ and long‐term outcome 1, 2. The factors that determine alveolar recovery or progressive fibrosis are unclear.

MV may increase oxidative stress in the pathogenesis of ALI and is a potent stimulus for the production of TGF‐β1 3. As a major profibrogenic cytokine, TGF‐β1 is crucial in the transdifferentiation of epithelial cells into myofibroblasts‐like cells—a phenomenon termed EMT—which contributes to the fibroproliferative response in patients with ARDS 3, 6. This transition features the loss of epithelial markers (E‐cadherin and aquaporin‐5) and apical–basal polarity as well as cytoskeletal rearrangement, transition to a spindle‐shaped morphology and the acquisition of mesenchymal markers (α‐smooth muscle actin (α‐SMA) and N‐cadherin) 7, 8. Furthermore, MV can induce EMT and thus initiate and propagate VILI‐associated lung fibrosis 8, 9, 10. The Src protein tyrosine kinase is expressed by leucocytes, alveolar epithelial cells, endothelial cells and fibroblasts in the lung. It is a non‐receptor tyrosine kinase that is crucial for intracellular signal transduction related to cell proliferation, migration, differentiation, adhesion and apoptotic cell death 11, 12. A previous murine study demonstrated that MV can augment bleomycin‐induced EMT through the Src pathway 13. The profibrotic effect of Src kinases could be an attractive target in the management of pulmonary fibrosis by using current novel tyrosine kinase inhibitors 14, 15, 16.

Nintedanib is an intracellular inhibitor of tyrosine kinases that targets platelet‐derived growth factor (PDGF) receptors α/β, fibroblast growth factor (FGF) receptors 1–3, vascular endothelial growth factor (VEGF) receptors 1–3, TGF‐β, c‐Abelson and Src family kinases. Nintedanib exhibits significant therapeutic effects on modulating myofibroblast differentiation and extracellular matrix (ECM) secretion *in vitro* and attenuating bleomycin‐ and silica‐induced pulmonary fibrosis in animal models 17, 18, 19, 20, 21, 22. Nintedanib is also an antifibrotic agent that—on the basis of clinical studies that showed that it slowed the rate of disease progression in lung function—has been approved by the Food and Drug Administration for idiopathic pulmonary fibrosis (IPF) management in the Unites States and Europe 23, 24. However, the strategy directly aimed at attenuating the fibroproliferative activity of ARDS is still lacking and the clinical application of nintedanib as an effective antifibrotic agent in this acute critical situation is hopefully anticipated.

Using mice pre‐treated with bleomycin as a VILI model to simulate the early fibroproliferative response in clinical ARDS, we first explored whether nintedanib could inhibit MV‐augmented bleomycin‐induced EMT and pulmonary fibrosis. Further, we compared the effects of various tidal volumes of MV and examined the role of Src kinases in the anti‐inflammatory, antifibrotic and anti‐apoptotic effects of nintedanib using Src‐deficient mice. We suggested that, following bleomycin‐induced ALI, nintedanib therapy can ameliorate high tidal volume mechanical stretch‐augmented EMT and pulmonary fibrosis by suppressing the Src pathway.

## Materials and methods

### Ethics of experimental animals

Wild‐type or Src‐deficient C57BL/6 mice, aged between 6 and 8 weeks, weighing between 20 and 25 g, were obtained from Jackson Laboratories (Bar Harbor, ME, USA) and National Laboratory Animal Center (Taipei, Taiwan), as described in our previous study 13. We performed the experiments in accordance with the National Institutes of Health Guidelines on the Use of Laboratory Animals. The Institutional Animal Care and Use Committee of Chang Gung Memorial Hospital approved the protocol (Permit Number: 2015101201).

### Experimental groups

Animals were randomly distributed into eight groups in each experiment: group 1, control, non‐ventilated wild‐type mice without bleomycin; group 2, control, non‐ventilated wild‐type mice with bleomycin; group 3, low tidal volume (V_T_ 6 ml/kg) wild‐type mice with bleomycin; group 4, high tidal volume (V_T_ 30 ml/kg) wild‐type mice with bleomycin; group 5, V_T_ 30 ml/kg Src ^+/−^ mice with bleomycin; group 6, V_T_ 30 ml/kg wild‐type mice with bleomycin and 100 mg/kg/day nintedanib administration; group 7, V_T_ 30 ml/kg wild‐type mice with bleomycin and 60 mg/kg/day nintedanib administration; group 8, V_T_ 30 ml/kg wild‐type mice with bleomycin and 30 mg/kg/day nintedanib administration. In groups 1–6, three mice underwent transmission electron microscopy (TEM) and five mice underwent measurement for Evans blue dye (EBD) assay, lung oedema, bronchoalveolar lavage (BAL) fluid total protein, malondialdehyde (MDA), TGF‐β1 production, Masson's trichrome stain, total collagen content, collagen gene expression, immunofluorescence labelling, fibrosis scoring, Western blot, immunohistochemistry assay, terminal deoxynucleotidyl transferase‐mediated dUTP‐biotin nick end‐labelling (TUNEL) assay and electron microscopy. In groups 7 and 8, five mice underwent measurement for EBD assay, lung oedema, BAL fluid total protein, MDA, TGF‐β1 production, Masson's trichrome stain, total collagen content, collagen gene expression, immunofluorescence labelling, fibrosis scoring, Western blot and immunohistochemistry assay.

### Ventilator protocol

We used our established mouse model of VILI, as previously described 13. In brief, a 20‐gauge angiocatheter was introduced into the tracheotomy orifice of mice and general anaesthesia was maintained by regular intraperitoneal administration of zoletil 50 (5 mg/kg) and xylazine (5 mg/kg) at the beginning of experiment and every 30 min. The mice were placed in a supine position on a heating blanket and then attached to a Harvard apparatus ventilator, model 55‐7058 (Harvard Apparatus, Holliston, MA, USA), set to deliver either 6 ml/kg at a rate of 135 breaths per min or 30 ml/kg at a rate of 65 breaths per min, for 5 hrs while breathing room air with zero end expiratory pressure. The tidal volume delivered by the ventilator was checked by fluid displacement from an inverted calibration cylinder. Continuous monitoring of end‐tidal CO_2_ by a microcapnograph (Columbus Instruments, Columbus, OH, USA) was performed, and respiratory frequencies of 135 breaths per min for 6 ml/kg and 65 breaths per min for 30 ml/kg were chosen in the experiment, with end‐tidal CO_2_ at 30–40 mmHg. Airway peak inspiratory pressure was measured with a pressure‐transducer amplifier (Gould Instrument Systems, Valley View, OH, USA) connected to the tubing at the proximal end of the tracheostomy. Mean arterial pressure was monitored every hour during MV using the same pressure‐transducer amplifier connected to a 0.61 mm outer diameter (0.28 mm inner diameter) polyethylene catheter ending in the common carotid artery. At the end of the study period, heparinized blood was taken from the arterial line for analysis of arterial blood gas, and the mice were killed. The non‐ventilated control mice were anaesthetized and killed immediately.

### Bleomycin and nintedanib administration

Nintedanib was administered once daily through gavage at 30, 60 and 100 mg/kg for 5 days before MV (Boehringer Ingelheim, Biberach, Germany) 21. The mice received a single dosage of 0.075 units of bleomycin in 100 μl of sterile normal saline solution intratracheally (Sigma‐Aldrich, St. Louis, MO, USA) and were ventilated 5 days after the administration of bleomycin 13.

### Collagen assay

The lungs were homogenized, and collagen was solubilized in 0.5 M acetic acid. The protein extracts were incubated with Sirius red dye, and levels of collagen in lung tissues were determined at absorbance of 540 nm by SIRCOL collagen assay kit (Biocolor Ltd., Carrickfergus, County Antrim, UK) according to manufacturer's instructions. Amount of collagen was expressed in μg/g of wet lung weight.

### Immunoblot analysis

The lungs were homogenized in 3 ml of lysis buffer (20 mM HEPES pH 7.4, 1% Triton X‐100, 10% glycerol, 2 mM ethylene glycol‐bis (β‐aminoethyl ether)‐N, N, N′, N′‐tetraacetic acid, 50 μM β‐glycerophosphate, 1 mM sodium orthovanadate, 1 mM dithiothreitol, 400 μM aprotinin and 400 μM phenylmethylsulfonyl fluoride), transferred to Eppendorf tubes and placed on ice for 15 min. Tubes were centrifuged at 15,350× g for 10 min. at 4°C, and supernatant was flash‐frozen. Crude cell lysates were matched for protein concentration, resolved on a 10% bis‐acrylamide gel and electrotransferred to Immobilon‐P membranes (Millipore Corp., Bedford, MA, USA). For assay of Src, PDGFR, FGFR and VEGFR phosphorylation; Src total protein and glyceraldehydes‐phosphate dehydrogenase (GAPDH) expression, Western blot analyses were performed with antibodies of phospho‐Src, phospho‐PDGFRα/β, phospho‐FGFR1, phospho‐VEGFR2, Src and GAPDH (New England BioLabs, Beverly, MA, USA). Blots were developed by enhanced chemiluminescence (NEN Life Science Products, Boston, MA).

### Immunofluorescence labelling

The lung tissues were paraffin embedded, sliced at 4 μm, deparaffinized and stained according to the manufacturer's instruction for an immunohistochemical kit (Santa Cruz Biotechnology, Santa Cruz, CA, USA). Lung sections were incubated with primary rabbit antimouse antibodies of E‐cad and α‐SMA (1:100; New England BioLabs) and fluorescent secondary antibodies of FITC‐conjugated affinity purified anti‐goat (E‐cad) and Cy3‐conjugated anti‐rabbit (α‐SMA) (1:1000; Santa Cruz Biotechnology). To further determine the effects of MV on angiogenesis, lung sections were incubated with primary goat antimouse antibody of CD‐31 (1:1000; Santa Cruz Biotechnology) and fluorescent secondary antibody of Cy3‐conjugated anti‐goat (1:1000; Santa Cruz Biotechnology). Nuclear staining was performed using Hoechst solution (0.5 μg/ml; Sigma‐Aldrich). The fluorescence‐labelled slides were subsequently examined using a Leica TCS 4D confocal laser scanning microscopy system (Leica, Wetzlar, Germany).

### Terminal deoxynucleotidyl transferase‐mediated dUTP‐biotin nick end‐labelling Assay

The lung tissues were paraffin embedded, sliced at 4 μm and stained with a TUNEL labelling reaction mixture using TdT DNA fragment detection kit according to the manufacturer's instruction (Oncogen Research Products, Boston, MA, USA). The specimens were detected by diaminobenzidine (DAB) and counterstained by methyl green. A dark‐brown DAB signal indicated positive staining of apoptotic cells, whereas shades of blue–green to greenish tan signified non‐reactive cells. Apoptosis‐positive cells were quantified as the average number of epithelial cells with dark‐brown DAB signals per bronchiole, which were counted from 10 non‐overlapping fields by a single investigator blinded to therapeutic category of the mouse.

### Statistical evaluation

The Western blots were quantitated using a National Institutes of Health (NIH) image analyzer Image J 1.27z (National Institutes of Health, Bethesda, MD, USA) and presented as arbitrary units. Values were expressed as the mean ± S.D. from at least five separate experiments. The data of EBD analysis, lung wet‐to‐dry‐weight ratio, TGF‐β1, total collagen content, collagen gene expression, fibrosis score, histopathologic assay and oxygenation were analysed using Statview 5.0 (Abascus Concepts Inc. Cary, NC, USA; SAS Institute, Inc. Cary, NC, USA). All results of Western blots and real‐time PCR were normalized to the non‐ventilated control wild‐type mice with bleomycin treatment. anova was used to assess the statistical significance of the differences, followed by multiple comparisons with a Scheffe′s test, and a *P* value < 0.05 was considered statistically significant. BAL total protein, collagen gene expression, EBD analysis, immunohistochemistry, analysis of lung water, Masson's trichrome stain and fibrosis scoring, MDA, TEM, TUNEL and measurement of TGF‐β1 were performed as previously described 13.

## Results

### Reduction of VILI by nintedanib

**Figure 1:**
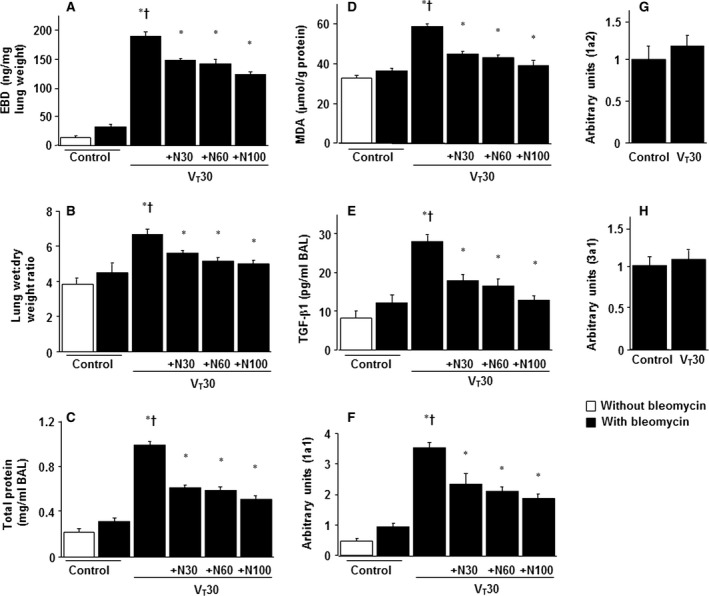
Nintedanib inhibited lung stretch‐induced microvascular leak, lung oedema, oxygen radicals, TGF‐β1 production and collagen gene expression. Five days after administering bleomycin, Evans blue dye analysis (**A**), lung wet‐to‐dry‐weight ratio (**B**), BAL fluid total protein (**C**), MDA (**D**) and TGF‐β1 secretion in BAL fluid (**E**) were from the lungs of non‐ventilated control mice and those subjected to tidal volume (V_T_) at 30 ml/kg (V_T_ 30) for 5 hrs with room air (*n* = 5 per group). Real‐time polymerase chain reaction of collagen 1a1 (**F**), 1a2 (**G**) and 3a1 (**H**) mRNA expression in lung tissue after 5 days of bleomycin administration was from the non‐ventilated control mice and those subjected to V_T_ at 30 ml/kg for 5 hrs with room air (*n* = 5 per group). Arbitrary units were expressed as the ratio of 1a1, 1a2 and 3a1 mRNA to GAPDH (*n* = 5 per group). Oral nintedanib was administered once daily in doses of 30, 60 and 100 mg/kg for 5 days before mechanical ventilation. *P < 0.05 *versus* the non‐ventilated control mice with bleomycin pre‐treatment; ^**†**^ *P* < 0.05 *versus* all other groups. BAL = bronchoalveolar lavage; EBD = Evans blue dye; GAPDH = glyceraldehydes‐ phosphate dehydrogenase; MDA = malondialdehyde; N30 = 30 mg/kg nintedanib; N60 = 60 mg/kg nintedanib; N100 = 100 mg/kg nintedanib; TGF‐β1 = transforming growth factor‐β1 (TGF)‐β1.

We applied high tidal volume and low tidal volume MV with room air for 5 hrs to induce VILI in mice and examined the injurious effects of overdistension and treatment effects of orally delivered nintedanib. The physiological conditions at the beginning and end of ventilation are listed in Table 1. The normovolemic statuses of the mice were maintained by monitoring their mean artery pressure. The lung EBD microvascular leak, wet‐to‐dry‐weight ratio and BAL fluid total protein were measured to determine the effects of high tidal volume ventilation on microvascular permeability and lung oedema in VILI (Fig. 1A–C and Fig. S1). Furthermore, the levels of oxidant loads and TGF‐β1 protein production were measured to determine the level of oxidative stress and amount of profibrogenic cytokines for fibroblasts in VILI (Fig. 1D and E). Elevated levels of EBD, wet‐to‐dry‐weight ratio, BAL fluid total protein, angiogenesis, MDA (an aldehydic secondary product of lipid peroxidation) and TGF‐β1 protein were observed in mice subjected to a tidal volume of 30 ml/kg compared with non‐ventilated control mice. We also used real‐time PCR to measure the expression of inflammation‐associated collagen 1a1, 1a2 and 3a1. Only collagen 1a1 expression was higher in mice subjected to V_T_ at 30 ml/kg compared with non‐ventilated control mice (Fig. 1F–H). The administration of nintedanib substantially and dose dependently suppressed the high tidal volume MV‐induced increase in lung inflammation and the collagen 1a1 expression.

**Table 1:** Physiological conditions at the beginning and end of ventilation

	Control without bleomycin wild‐type	Control with bleomycin wild‐type	V_T_ 6 ml/kg with bleomycin wild‐type	V_T_ 30 ml/kg with bleomycin wild‐type	V_T_ 30 ml/kg with bleomycin Src^+/−^	V_T_ 30 ml/kg with bleomycin wild‐type, N30	V_T_ 30 ml/kg with bleomycin wild‐type, N60	V_T_ 30 ml/kg with bleomycin wild‐type, N100
PH	7.42 ± 0.03	7.40 ± 0.04	7.34 ± 0.05	7.35 ± 0.04	7.39 ± 0.06	7.37 ± 0.04	7.38 ± 0.07	7.36 ± 0.06
PaO_2_ (mmHg)	97.9 ± 0.4	96.3 ± 0.2	85.4 ± 0.7*	76.8 ± 1.4* ^,^ †	86.5 ± 1.2*	79.2 ± 1.4*	80.7 ± 1.1*	84.2 ± 1.3*
PaCO_2_ (mmHg)	39.2 ± 0.1	39.0 ± 0.3	41.0 ± 0.2	36.3 ± 0.8	36.9 ± 1.4	37.7 ± 1.5	37.5 ± 1.6	36.8 ± 1.4
MAP (mmHg)								
Start	85.1 ± 0.8	83.0 ± 0.8	82.7 ± 1.2	82.1 ± 1.5	83.1 ± 1.2	82.7 ± 1.6	82.5 ± 1.7	82.8 ± 1.9
End	83.2 ± 0.5	81.2 ± 0.4	79.2 ± 1.4	73.4 ± 4.9* ^,^ †	78.9 ± 1.6*	75.2 ± 4.5*	75.6 ± 4.2*	76.4 ± 4.5*
PIP, mmHg								
Start			10.0 ± 0.9	23.8 ± 1.4	23.5 ± 1.6	23.6 ± 1.1	23.7 ± 1.4	23.8 ± 1.2
End			11.8 ± 0.7	28.6 ± 2.2	26.9 ± 1.5	27.9 ± 2.5	27.4 ± 2.1	27.2 ± 1.8

At the end of the study period, arterial blood gases and mean arterial pressure were obtained from the non‐ventilated control mice and mice subjected to tidal volume at 6 ml/kg or at 30 ml/kg for 5 hrs (*n* = 10 per group). The normovolemic statuses of mice were maintained by monitoring mean artery pressure. Data are presented as mean ± S.D. *P < 0.05 *versus* the non‐ventilated control mice with bleomycin pre‐treatment; ^**†**^ *P* < 0.05 *versus* all other groups. MAP, mean arterial pressure; N30 = 30 mg/kg/day nintedanib; N60 = 60 mg/kg/day nintedanib; N100 = 100 mg/kg/day nintedanib; PIP, peak inspiratory pressure; Src^+/−^, Src‐deficient mice; V_T_, tidal volume. The physiological data of the control groups were similar during the experiment and were used as the beginning data of ventilation.

### Suppression of mechanical ventilation‐induced production of collagen fibres and collagen 1a1 expression by nintedanib

**Figure 2:**
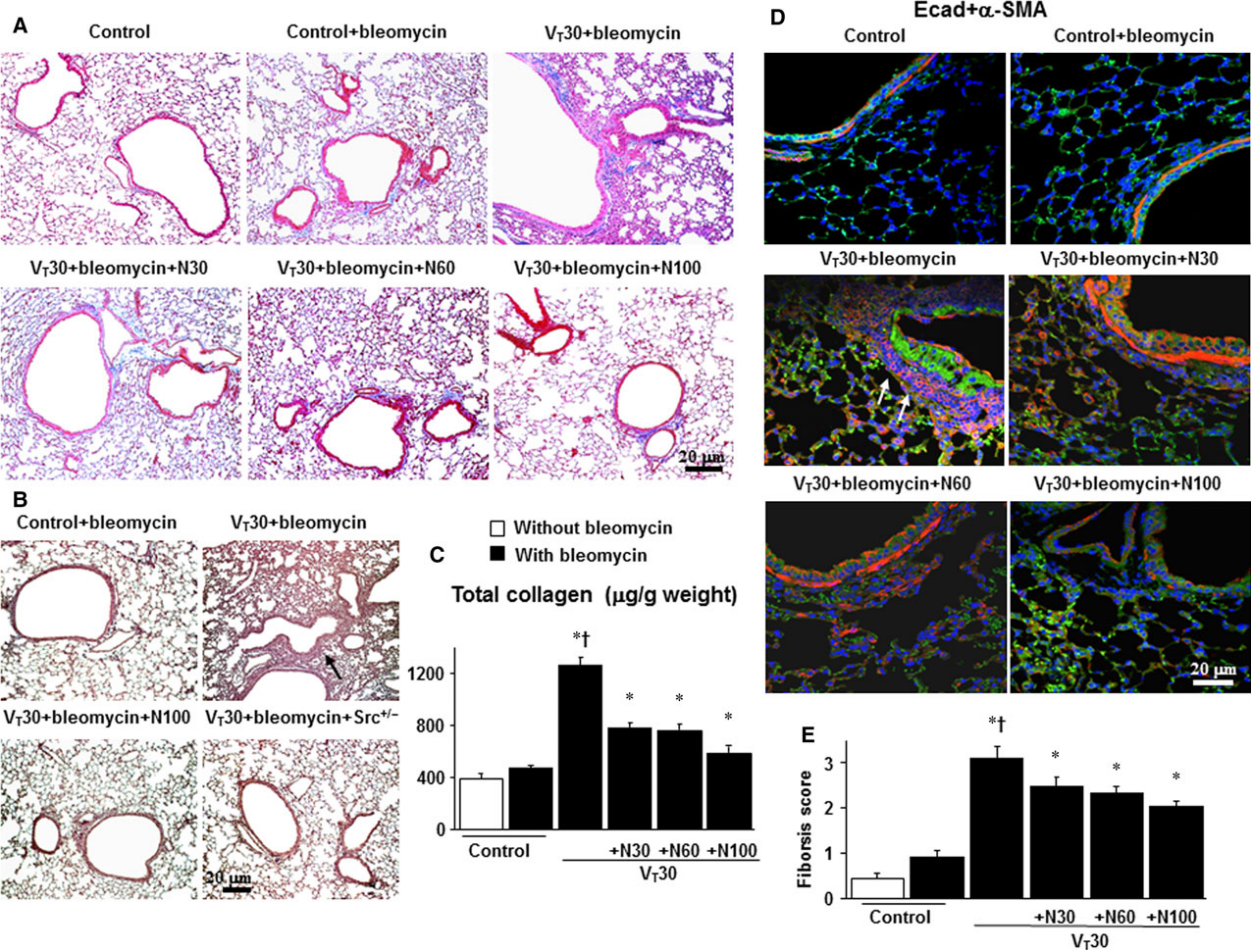
Nintedanib reduced lung stretch‐induced production of collagen and fibrogenic biomarkers. Representative micrographs (×100) with Masson's trichrome staining of paraffin lung sections (**A**); micrographs (×400) with an antibody that recognizes collagen 1a1 expression in paraffin lung sections (**B**); collagen of lung tissue (**C**); and photomicrographs (×400) with E‐cadherin (E‐cad, bright green), α‐smooth muscle actin (α‐SMA, red) and Hoechst (blue) immunofluorescent staining of paraffin lung sections (**D**) after 5 days of bleomycin treatment were from the non‐ventilated control mice and those subjected to V_T_ at 30 ml/kg for 5 hrs with room air (*n* = 5 per group). Oral nintedanib was administered once daily in doses of 30, 60 and 100 mg/kg for 5 days before mechanical ventilation. A dark‐brown diaminobenzidine signal identified by arrows indicates positive staining for collagen 1a1 in the lung epithelium or interstitial, whereas shades of bluish tan signify non‐reactive cells. Positive red staining in the lung epithelium and interstitium is identified by arrows (*n* = 5 per group). The fibrotic scoring (**E**) was quantified as the average number of 10 non‐overlapping fields in Masson's trichrome staining of paraffin lung sections (*n* = 5 per group). Scale bars represent 20 μm. *P < 0.05 *versus* the non‐ventilated control mice with bleomycin pre‐treatment; ^**†**^ *P* < 0.05 *versus* all other groups. Src^+/−^ = Src‐deficient mice.

We used Masson's trichrome staining to determine the effects of MV on the accumulated peribronchiolar and parenchymal collagen fibres. We observed increased collagen fibres in the ECM in mice subjected to V_T_ at 30 ml/kg compared with non‐ventilated control mice (Fig. 2A). Furthermore, we applied immunohistochemistry and observed that positively stained epithelium and parenchymal collagen fibres for collagen 1a1 were substantially increased in mice subjected to MV compared with non‐ventilated control mice (Fig. 2B). The increase in collagen fibres, measured quantitatively according to total lung collagen contents, was substantially and dose dependently attenuated by pharmacologic inhibition with nintedanib (Fig. 2C).

### Inhibition of mechanical ventilation‐induced fibrogenic markers by nintedanib

We measured the expression of E‐cadherin, an epithelial marker, and α‐SMA, a mesenchymal marker, through immunofluorescent staining to identify the cells types involved in the lung stretch‐induced EMT (Fig. 2D). Moreover, we explored the effects of nintedanib treatment in attenuating EMT. Mice subjected to V_T_ at 30 ml/kg demonstrated downregulation of E‐cadherin and upregulation of α‐SMA in the bronchiolar epithelium and peribronchiolar lung parenchyma, indicating a phenotype transition from epithelial cells to myofibroblasts. Nintedanib dose dependently increased the expression of E‐cadherin and decreased the expression of α‐SMA. To further determine the effects of MV on ECM accumulation, we performed fibrosis scoring through Masson's trichrome staining (Fig. 2A and E). The extent of peribronchiolar ECM deposition was higher in mice subjected to V_T_ at 30 ml/kg than in non‐ventilated control mice. Nintedanib administration substantially ameliorated the fibrosis score of ventilation‐induced lung fibrogenesis.

### Suppression of the mechanical ventilation‐induced Src pathway by nintedanib

**Figure 3:**
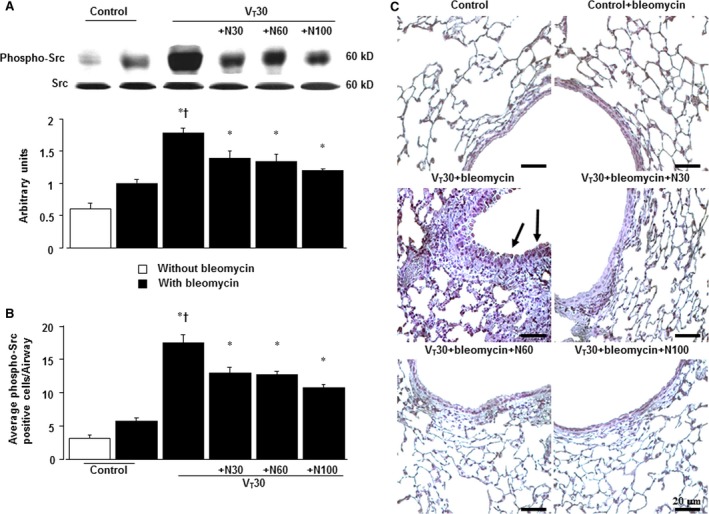
Nintedanib abrogated lung stretch‐induced Src phosphorylation. Western blot (**A**) conducted using an antibody that recognizes the phosphorylated Src expression and an antibody that recognizes total Src expression in lung tissue after 5 days of bleomycin treatment were from the non‐ventilated control mice and those subjected to V_T_ at 30 ml/kg for 5 hrs with room air (*n* = 5 per group). Arbitrary units were expressed as the ratio of phospho‐Src to Src (*n* = 5 per group). Representative micrographs (×400) with phosphorylated Src staining of paraffin lung sections and quantification (**B** and **C**) 5 days after administering bleomycin were from the non‐ventilated control mice and those subjected to V_T_ at 30 mL/kg for 5 hrs with room air (*n* = 5 per group). Oral nintedanib was administered once daily in doses of 30, 60 and 100 mg/kg for 5 days before mechanical ventilation. A dark‐brown diaminobenzidine signal identified by arrows indicates positive staining for phospho‐Src in the lung epithelium or interstitial, whereas shades of bluish tan signify non‐reactive cells. Scale bars represent 20 μm. *P < 0.05 *versus* the non‐ventilated control mice with bleomycin pre‐treatment; ^**†**^ *P* < 0.05 *versus* all other groups.

Because Src activation has been reported to regulate stretch‐induced EMT, we measured Src phosphorylation to investigate the role of the Src pathway in our VILI model (Fig. 3) 11, 13. Western blot analyses revealed increased Src phosphorylation in mice subjected to MV with room air compared with non‐ventilated control mice. Moreover, the administration of nintedanib dose dependently attenuated MV‐induced phospho‐Src activation (Fig. 3A). The expression of the total Src non‐phosphorylated proteins did not change significantly. Consistent with the results of Western blot, positive results for immunohistochemical staining for Src in the bronchial epithelium of mice subjected to V_T_ at 30 ml/kg was dose dependently attenuated by nintedanib treatment (Fig. 3B).

### Inhibition of mechanical ventilation‐induced lung inflammation and EMT by Src heterozygous knockout

**Figure 4:**
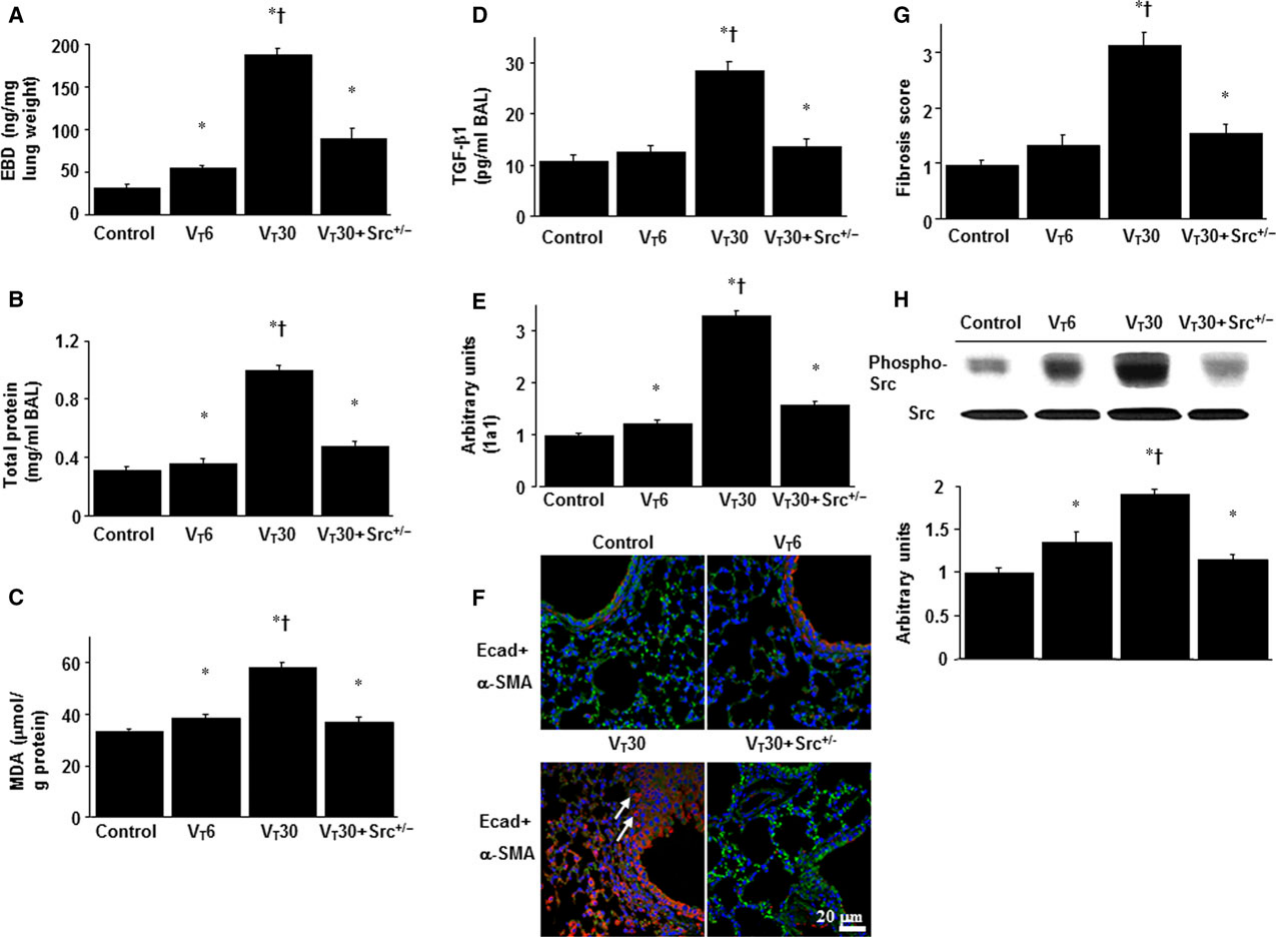
Inhibition of lung stretch‐induced lung inflammation, epithelial–mesenchymal transition (EMT) and Src phosphorylation by Src heterozygous knockout. Five days after administering bleomycin, Evans blue dye analysis (**A**), BAL fluid total protein (**B**), MDA (**C**), TGF‐β1 secretion in BAL fluid (**D**) and collagen 1a1 expression (**E**) were from the lungs of non‐ventilated control mice and those subjected to tidal volume at 6 ml/kg (V_T_ 6) or at 30 ml/kg for 5 hrs with room air (*n* = 5 per group). Arbitrary units were expressed as the ratio of 1a1 mRNA to GAPDH (*n* = 5 per group). Representative photomicrographs (×400) with E‐cad (bright green), α‐SMA (red) and Hoechst (blue) immunofluorescent staining of paraffin lung sections (**F**) 5 days after administering bleomycin were from the lung tissue of non‐ventilated control mice and those subjected to V_T_ at 6 ml/kg or at 30 ml/kg for 5 hrs with room air (*n* = 5 per group). Positive red and bright green staining in the fibroblasts is identified by arrows (*n* = 5 per group). The fibrotic scoring (**G**) was quantified as the average number of 10 non‐overlapping fields in Masson's trichrome staining of paraffin lung sections (*n* = 5 per group). Western blot (**H**) conducted using an antibody that recognizes the phosphorylated Src expression and an antibody that recognizes total Src expression in lung tissue 5 days after bleomycin treatment were from the non‐ventilated control mice and those subjected to V_T_ at 6 ml/kg or at 30 ml/kg for 5 hrs with room air (*n* = 5 per group). Arbitrary units were expressed as the ratio of phospho‐Src to Src (*n* = 5 per group). Scale bars represent 20 μm. *P < 0.05 *versus* the non‐ventilated control mice with bleomycin pre‐treatment; ^**†**^ *P* < 0.05 *versus* all other groups.

We compared the effects of various tidal volume of MV (V_T_ 30 ml/kg *versus* V_T_ 6 ml/kg) to examine the effect of lung‐protective ventilator strategy on the Src signalling pathway in VILI. Furthermore, we employed Src‐deficient mice to determine whether the beneficial effects provided by the administration of nintedanib were mediated through the Src pathway. The effects of high tidal volume lung stretch on increase in lung EBD leak, BAL fluid total protein, oxidative stress, TGF‐β1 protein production, collagen 1a1 expression, fibrogenic markers, fibrosis scoring and Src phosphorylation in mice subjected to V_T_ at 30 ml/kg were substantially attenuated in Src‐deficient mice and those subjected to V_T_ at 6 ml/kg (*P* < 0.05, Fig. 4).

There was only partial inhibition of lung injury and fibrogenesis through Src knockout and pharmacologic inhibition with nintedanib suggested that there are other potential mechanisms, such as PDGFR, FGFR and VEGFR pathways contributing to MV‐induced EMT and pulmonary fibrosis (Fig. S2). Because no statistically significant differences were observed between wild‐type and Src‐deficient non‐ventilated control mice with or without bleomycin, the data are not presented in this article.

### Reduction in mechanical ventilation‐induced epithelial apoptosis by Src heterozygous knockout and nintedanib

**Figure 5:**
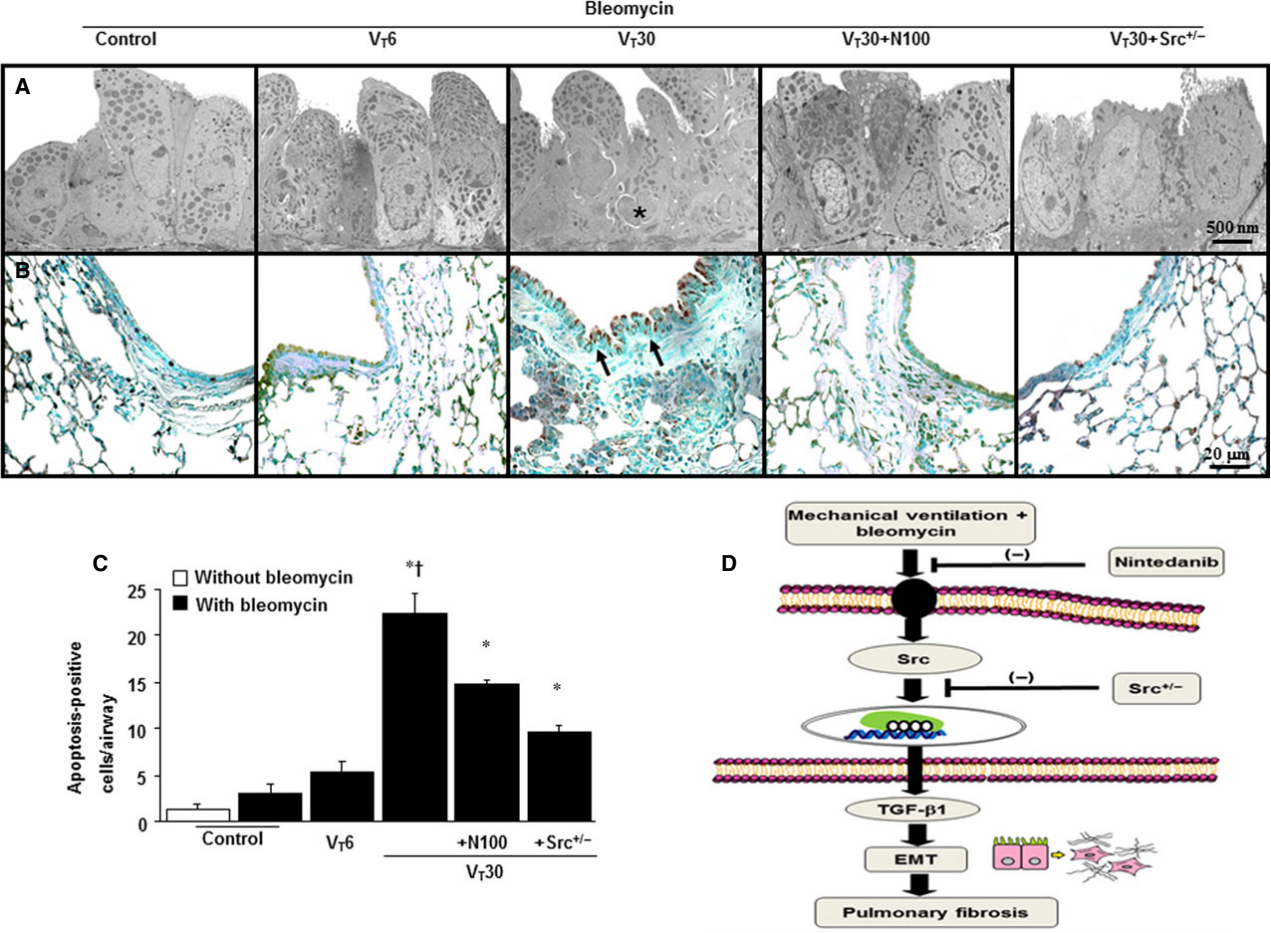
Src heterozygous knockout and nintedanib attenuated lung stretch‐induced bronchial epithelial apoptosis**.** Five days after bleomycin treatment, representative micrographs with transmission electron microscopic image (**A**, ×6000) (*n* = 3 per group) and TUNEL staining (**B** and **C**, ×400) (*n* = 5 per group) of the paraffin lung sections were from non‐ventilated control mice and those subjected to V_T_ at 6 ml/kg or at 30 ml/kg for 5 hrs with room air. Oral nintedanib was administered once daily in a dose of 100 mg/kg for 5 days before mechanical ventilation. Highly condensed and fragmented heterochromatin of bronchial epithelial cells indicates apoptosis. A dark‐brown diaminobenzidine signal indicated positive staining of apoptotic cells, whereas shades of blue–green to greenish tan signified non‐reactive cells. Apoptotic cells are identified by asterisks or arrows. (**D**) Schematic figure: bleomycin‐induced augmentation of mechanical stretch‐mediated cytokine production and EMT were attenuated by the administration of nintedanib and with Src heterozygous knockout. Scale bars represent 500 nm or 20 μm. *P < 0.05 *versus* the non‐ventilated control mice with bleomycin pre‐treatment; ^**†**^ *P* < 0.05 *versus* all other groups. EMT = epithelial–mesenchymal transition; TUNEL = terminal deoxynucleotidyl transferase‐mediated dUTP‐biotin nick end‐labelling.

Because Src activation has been associated with MV‐induced pathway‐driven lung inflammation, we performed TEM and TUNEL staining to determine the effects of Src deficiency in mice and the influence of nintedanib on ventilation‐induced apoptosis of airway epithelial cells (Fig. 5A–C). Epithelial apoptosis was confirmed by the characteristic nuclear condensation of bronchial epithelium in mice receiving high tidal volume MV. The increases of V_T_30‐induced epithelial apoptosis were attenuated after Src heterozygous knockout and the administration of nintedanib. Collectively, our results demonstrated that nintedanib therapy can suppress MV‐induced inflammatory responses and EMT in the lung by inhibiting the Src pathway (Fig. 5D).

## Discussion

ARDS are disorders of acute hypoxemic respiratory failure, characterized by increased microvascular permeability and pulmonary oedema 1. Previous studies have suggested that elevated amounts of inflammatory cytokines, increased numbers of mesenchymal cells and collagen accumulation in the interstitium might occur in the reparative phase of ARDS. This decreases pulmonary compliance and causes the severe hypoxaemia associated with the high morbidity and mortality of patients with ARDS 1, 2, 3, 4, 5. Clinical research has highlighted the importance of early fibroproliferation in the lungs of patients who experience ARDS; 47% of patients had computed tomography evidence of pulmonary fibrosis as early as the first day of ARDS, whereas TGF‐β1 and collagen were detected in BAL fluid as early as 24 hrs after the diagnosis of ARDS 3, 4, 5. Survivors of ARDS developed irreversible pulmonary fibrosis, leading to a reduced quality of life and poor prognosis 1.Therefore, early intervention that targets fibroproliferative activity to ameliorate the progression of subsequent pulmonary fibrosis may improve clinical outcome in ARDS patients. A previous murine model of acid‐induced lung injury demonstrated that 2 hrs of MV induced lung fibrogenesis associated with increased TGF‐β1 production 7. Our previous studies of VILI in mice revealed that 5 hrs of high tidal volume MV induced the production of hyaluronan, plasminogen activator inhibitor‐1 and TGF‐β1 in fibroblasts, leading to ECM‐induced inflammatory changes and EMT 13, 25. Fibroblast proliferation and extracellular matrix accumulation are initiated 4‐14 days after bleomycin challenge 26. Previous studies have demonstrated that angiogenic activities and histopathologic evidence of fibroproliferation were found as early as 5 days in the course of ARDS and in a mouse model of pulmonary fibrosis induced by bleomycin 27, 28. Therefore, 5 days after administering bleomycin, then exposure of mice to 5 hrs of MV was used in our study to focus on the major target involved in the early‐phase of fibroproliferation after ALI. In this injurious MV model using mice after bleomycin exposure, we observed that high tidal volume MV increased lung oedema, microvascular permeability, collagen deposition, EMT, epithelial apoptosis and TGF‐β1 production. The Src pathway regulated the increase of lung inflammation and pulmonary fibrosis.

In addition to playing a critical role in the pathogenesis of MV‐ and oxidative stress‐related ARDS, TGF‐β1 enhances fibroblast recruitment and ECM deposition and might induce cytoskeletal remodelling that lead to progressive fibrosis 19, 29. Mishra R *et al*. revealed that TGF‐β1‐mediated type I collagen synthesis, assembly and secretion are dependent on the activation of Src in human mesangial cells 16. Collagens are the most abundant protein of the ECM and maintain the normal lung architecture. Type I collagen is more prevalent in the late‐phase of ALI, whereas type III collagen fibre, which is relatively more flexible and susceptible to breakdown, predominates in the early stage of ALI 20, 30, 31, 32, 33. Furthermore, mechanical forces can modify the gene expression and structural remodelling of ECM through increased transpulmonary pressure, heterogeneous distribution of ventilation, increased tissue stretch, reduced pulmonary lymphatic drainage and direct secretion of various growth factors, including TGF‐β1 9. We demonstrated that high tidal volume MV increased oxidative stress, collagen accumulation and increase of collagen 1a gene expression. Moreover, epithelial cells can promote pulmonary fibrogenesis by acquiring a TGF‐β1‐mediated mesenchymal phenotype 6. Myofibroblasts are the predominant effective mesenchymal cells involved in pulmonary fibrogenesis and can originate from EMT of abnormally activated alveolar epithelium 34. Local increase of TGF‐β1, high tidal volume mechanical stretch and the presence of the extra domain A splice variant of fibronectin are three major factors driving the differentiation of fibroblasts to myofibroblasts 35. Additionally, a previous A549 epithelial cell study showed that both the transcript and protein expressions of E‐cadherin substantially increased following nintedanib treatment, indicating that EMT reversal can be achieved by tyrosine kinase inhibition 36. In the current study of high tidal volume mechanical stretch after bleomycin‐induced ALI, we further identified that nintedanib treatment dose dependently increased the expression of E‐cadherin and decreased the expression of α‐SMA (Fig. 2D).

At present, no specific pharmacological reagents effectively prevent early fibroproliferative activity in ARDS. The use of corticosteroids may shorten the duration of MV, but the optimal timing and duration for their administration remain undetermined and their influence on enhancing ARDS survival is unproven 37, 38, 39. Previous clinical trials (INPULSIS‐1 and INPULSIS‐2) have evaluated the efficacy and safety of nintedanib in patients with IPF and have revealed that nintedanib therapy ameliorated the decline in forced vital capacity, which is consistent with a delay in disease progression 19, 23. Nintedanib, an indolinone derivative originally designed as an anti‐angiogenic drug, targets the intracellular ATP binding pockets of PDGF, FGF and VEGF receptors. It also inhibits TGF‐β1‐induced fibroblast to myofibroblast differentiation and hinders non‐receptor tyrosine kinases of the Src family in bleomycin murine lung fibrosis models 15, 22, 40. The Src tyrosine kinase has been proven to play a pivotal role in the regulation of both proinflammatory and profibrotic responses in ALI 11, 12, 15. TGF‐β1 can induce the activation of Src by autophosphorylation of the tyrosine 416 41. In the presence of active TGF‐β1, during a murine study of bleomycin‐induced lung fibrosis, epithelial integrin and focal adhesion kinases up‐regulated Src activity and Src inhibition attenuated EMT, which is a potential source of myofibroblasts accumulation in fibrotic lungs 42, 43, 44. Moreover, previous studies have demonstrated that oral administration of 60 and 100 mg/kg of nintedanib is sufficient to inhibit FGFRs, VEGFRs, Lck, Src and Flt‐3 in mice 21, 40. In the present study, we demonstrated that genetic downregulation of Src prevented the increase of TGF‐β1, EMT, collagen 1a1 and lung fibrosis scores through the inhibition of Src activation. Thus, the profibrotic effects of Src kinase provide a promising therapeutic option in the management of VILI‐associated pulmonary fibrosis. Furthermore, we examined the effects of nintedanib on MV‐augmented bleomycin‐mediated Src activation, EMT and pulmonary fibrosis.

High tidal volume MV can enhance Src signalling by activating adherens junctions, Ca^2+^ entry through stretch‐activated cation channels, focal adhesion kinases and G protein‐coupled receptors as well as remodelling cytoskeleton and integrins 45. Mechanical stretch‐induced Src activation in rats has been proven to augment intracellular signal transduction and the dissociation of junctional proteins from their cytoskeletal anchors, which may cause endothelial gap formation and an increase in vascular permeability 46. Previous studies of mouse embryonic fibroblasts and bleomycin‐induced murine lung fibrosis models indicated that selective Src kinase inhibition using PP2, a Src kinase inhibitor, reduced myofibroblast differentiation and pulmonary fibrosis 13, 14. Blocking the Src pathway is likely to have some beneficial effects on the epithelial integrity, which may contribute to the reduced lung fibrosis. However, the protective effects of nintedanib on VILI‐associated EMT and pulmonary fibrosis are not well defined. Nintedanib can inhibit the proliferation, migration, differentiation to myofibroblasts and TGF‐β1‐induced collagen secretion in primary human lung fibroblasts 19, 21. In animal models of both bleomycin‐induced and silica‐enhanced pulmonary fibrosis, reduced leucocyte counts, cytokine levels and total lung collagen as well as improving pathologic alterations showed that nintedanib treatment can inhibit pulmonary inflammation and fibrosis 21. In this study, we demonstrated that nintedanib, a multitargeted tyrosine kinase inhibitor, magnitude dependently suppressed high tidal volume MV‐induced Src phosphorylation and EMT (downregulation of an epithelial marker E‐cadherin and upregulation of a mesenchymal marker α‐SMA in the bronchiolar epithelium and peribronchiolar lung parenchyma), mimicking the inhibitory effects provided by Src heterozygous knockout (Fig. 4). Additionally, the lesional effect of MV on apoptosis may vary with cell types; TGF‐β1 was shown to enhance the apoptotic death of epithelium by activating the Fas/Fas ligand pathway 27. Nevertheless, no specific role in the epithelial apoptosis of pulmonary fibrosis has been described for nintedanib. In the present study, we observed that MV induced characteristic apoptosis of murine airway epithelial cells and that this can be attenuated by either nintedanib treatment or Src heterozygous knockout. These data indicate the involvement of the Src pathway in regulating EMT, pulmonary fibrosis and epithelial apoptosis.

Our study had some limitations. Compared with the control mice, partial inhibition of lung injury and fibrogenesis through Src knockout or pharmacologic inhibition with nintedanib suggested that Src signalling was only one of the many pathways contributing to MV‐induced EMT and pulmonary fibrosis. A previous human fibroblast study also indicated that nintedanib attenuated type II TGF‐β receptor‐activated ECM protein production, collagen 1a1 gene expression, Smad3 and p38 MAPK pathways 20. Additional experiments are necessary to explore the cross talk between Src and other signalling pathways, such as phosphoinositide 3‐OH kinase, serine/threonine kinase/protein kinase B and MAPK 45.

Using an *in vivo* murine model of bleomycin‐induced VILI, we demonstrated that nintedanib attenuated high tidal volume MV‐induced lung inflammation‐associated EMT, pulmonary fibrosis and epithelial apoptosis through the inhibition of Src signalling pathway and TGF‐β1 production. Though the ARDS network's clinical trials have shown that low tidal volume MV is safer than high tidal volume MV, the mortality of patients with ARDS has remained considerably high; the mechanisms of systemic translocation and multisystem organ failure related to ALI need to be further explored 47, 48. Knowledge of the effects of mechanical stretch on Src signalling allowed clarification of the pathophysiological mechanisms regulating the organic phase of ARDS, which can lead to irreversible pulmonary fibrogenesis and necessitate ventilator support. Therefore, inhibiting Src with nintedanib, a potent tyrosine kinase modulator, might serve as a novel therapeutic target for prevention and treatment of ARDS. Further identifying the mechanisms regulating the early reparative phase of ARDS is crucial for developing more effective management of various types of ALI.

## Conflicts of interest

The authors confirm that there are no conflict of interests.

## Author's contribution

L.F.L. and Y.Y.L. performed the experiments and wrote the manuscript. C.W.L., K.C.K., N.H.C., C.S.L., L.P.C. and C.T.Y. designed the experiments and analysed the data.

## Supporting information


**Figure S1** Inhibition of lung stretch‐induced angiogenesis by nintedanib and Src heterozygous knockout.

**Figure S2** Effects of nintedanib and Src heterozygous knockout on the mechanical ventilation‐induced PDGFR, FGFR, and VEGFR pathways.
