# Supplementary figures and images for: Nintedanib reduces ventilation‐augmented bleomycin‐induced epithelial–mesenchymal transition and lung fibrosis through suppression of the Src pathway
Source: J Cell Mol Med. 2017 Jun 9;21(11):2937–49. doi: 10.1111/jcmm.13206 (PMC5661114; doi:10.1111/jcmm.13206)

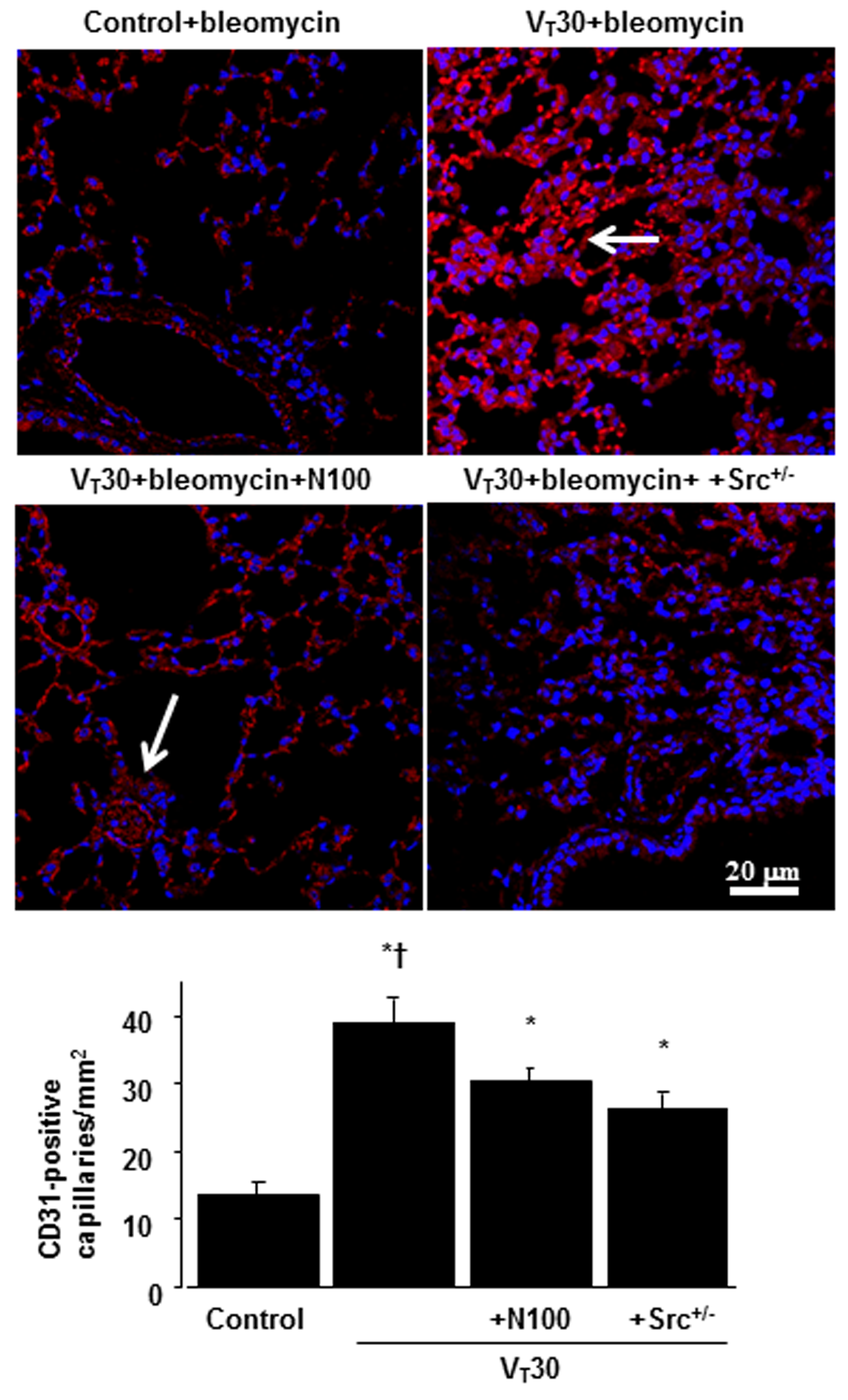

Supplement: Supplementary file 1 — Figure S1 Inhibition of lung stretch‐induced angiogenesis by nintedanib and Src heterozygous knockout. [file JCMM-21-2937-s001.tif]
