# Supplementary material for: Nintedanib reduces ventilation‐augmented bleomycin‐induced epithelial–mesenchymal transition and lung fibrosis through suppression of the Src pathway
Source: J Cell Mol Med. 2017 Jun 9;21(11):2937–49. doi: 10.1111/jcmm.13206 (PMC5661114; doi:10.1111/jcmm.13206)
Supplement: Supplementary file 3 [file JCMM-21-2937-s003.doc]

**Figure S1. Inhibition of lung stretch-induced angiogenesis by nintedanib and Src heterozygous knockout.** Representative photomicrographs (x400) with CD31 (red) and Hoechst (blue) immunofluorescent staining of paraffin lung sections after five days of bleomycin treatment were from the nonventilated control mice and those subjected to V_T_ at 30 mL/kg for 5 h with room air (n = 5 per group). Oral nintedanib was administered once daily in doses of 100 mg/kg for 5 days before mechanical ventilation. Positive red staining in the lung epithelium and interstitium is identified by arrows. Microvessels were counted in at least three random fields under a confocal microscope. The CD31-positive capillaries were counted and the microvessel density was expressed as counts per mm^2^ (n = 5 per group). Scale bars represent 20 μm. * P< 0.05 versus the nonventilated control mice with bleomycin pretreatment; **†** P< 0.05 versus all other groups. N100= 100 mg/kg nintedanib; Src^+/-^ = Src-deficient mice; V_T_ = tidal volume.

**Figure S2. Effects of nintedanib and Src heterozygous knockout on the mechanical ventilation-induced PDGFR, FGFR, and VEGFR pathways**. We measured PDGFR, VEGFR, and FGFR phosphorylation to examine the roles of the PDGFR, VEGFR, and FGFR pathways in our VILI model. Western blot analyses revealed increased PDGFR and VEGFR phosphorylation but decreased FGFR phosphorylation in mice subjected to MV with room air compared with nonventilated control mice. Reduced VEGFR phosphorylation but increased FGFR phosphorylation was observed after the administration of nintedanib and in Src-deficient mice. The mechanical ventilation**-**induced PDGFR phosphorylation was attenuated by nintedanib but not by Src heterozygous knockout. Further investigation is required to explore the cross talk between Src and other signaling pathways. Western blots using antibodies that recognize phosphorylated PDGFR (A), phosphorylated FGFR (B), phosphorylated VEGFR (C), and GAPDH expression in lung tissue after five days of bleomycin treatment were performed from the nonventilated control mice and those subjected to V_T_ at 30 mL/kg for 5 h with room air (n = 5 per group). Arbitrary units were expressed as the ratio of phospho-PDGFR, phospho-FGFR, and phospho-VEGFR to GAPDH (n = 5 per group). Oral nintedanib was administered once daily in doses of 100 mg/kg for 5 days before mechanical ventilation. * P< 0.05 versus the nonventilated control mice with bleomycin pretreatment; **†** P< 0.05 versus all other groups. FGFR = fibroblast growth factor receptors; GAPDH = glyceraldehydes- phosphate dehydrogenase; PDGFR = platelet-derived growth factor receptors; VEGFR = vascular endothelial growth factor receptors.
